# Diet and nutrition key factors for oral microbiota composition: a systematic review

**DOI:** 10.29219/fnr.v69.11956

**Published:** 2025-11-24

**Authors:** Heriberto Castro García, Vania Urias Orona, Luis Fernando Méndez, Andrea Arreguin Coronado, Marcela Alejandra Gloria Garza

**Affiliations:** 1 Centro de Investigación en Nutrición y Salud Pública, Facultad de Salud Pública y Nutrición, Universidad Autonoma de Nuevo León, Monterrey, Nuevo León, México; 2 Facultad de Enfermería y Nutrición, Universidad Autonoma de San Luis Potosí, San Luis Potosí, México; 3 Laboratorio de Microbiología, Facultad de Odontología, Universidad Autonoma de Nuevo León, Monterrey, Nuevo León, México

**Keywords:** oral microbiota, prebiotics, probiotics, sweeteners, dental health, DPs

## Abstract

**Background:**

Diet and nutrition are essential in preserving oral health as they influence the microbiota balance and modulate inflammatory responses. The relationship between dietary patterns (DPs) and oral diseases such as caries and periodontitis has garnered significant scientific attention. Objective. This review aims to analyze the impact of various DPs, nutrients, and supplementation with probiotics and prebiotics on oral health and microbiota modulation.

**Design:**

A systematic search was conducted in PubMed, Scopus, and Web of Science databases for clinical studies published between 2010 and 2024. A total of 514 articles were retrieved, 78 were evaluated at the full-text level, and 22 original clinical studies were included based on eligibility criteria. These studies were grouped into three categories: dietary interventions without supplementation, probiotic/prebiotic supplementation, and combined strategies.

**Results:**

Healthy DPs, such as the Mediterranean and vegetarian diets, showed anti-inflammatory effects and positively modulated oral microbiota by reducing pathogenic species linked to oral diseases. In contrast, high sugar consumption was associated with increased acidogenic bacterial species and dental caries. Probiotics, particularly strains of *Lactobacillus* and *Bifidobacterium*, demonstrated therapeutic benefits in managing gingivitis, halitosis, and caries, while prebiotics supported the growth of beneficial bacteria, complementing probiotic efficacy.

**Discussion:**

Nutritional interventions modulate oral microbiota by shifting microbial profiles toward eubiosis and attenuating inflammatory responses. The effectiveness of probiotics appears to be strain-dependent and may vary with host conditions, while prebiotics support their colonization and metabolic activity.

**Conclusion:**

Dietary interventions directly influence oral health by modulating microbiota composition and inflammatory responses. Probiotics show clinical promise, though their long-term, strain-specific effects need further study. Prebiotics may enhance these benefits by supporting probiotic activity. A diet rich in plant-based foods and bioactive compounds, combined with targeted supplementation, represents a viable strategy to promote oral health and microbiota balance.

## Popular scientific summary

- Diet plays a critical role in oral health.
- Variations in dietary macronutrients and diet types can shift the oral microbiota, resulting in oral disease like dental caries and periodontitis.
- This review provides evidence relationship between dietary and nutritional factors and oral bacterial composition.

The World Dental Federation (FDI) describes oral health as a multifaceted concept involving the ability to perform essential functions such as speaking, smiling, tasting, smelling, touching, chewing, and swallowing, as well as expressing a wide range of emotions confidently and without pain, discomfort, or disease. Poor oral health, including functional issues such as broken or missing teeth or poorly fitting dentures, can cause oral pain and infections, significantly impairing the quality of life. These issues often result in decreased nutrient intake, altered food choices, communication challenges, and problems with chewing and swallowing (1). Oral health is essential to overall health and is closely linked to nutritional status and general well-being, directly influencing patients’ quality of life and health outcomes (2).

Diet plays a critical role in oral health, with both the physical and chemical properties of foods and how they are consumed (such as eating frequency and method) affecting the teeth and supporting tissues. Proper nutrition supports the healthy development of teeth and gums and lowers the risk of certain oral diseases. Macronutrients and micronutrients influence oral health by contributing to tooth development, enamel and dentin synthesis, dental mineralization, and the activation of protective mechanisms (2).

Oral diseases such as dental caries and periodontitis are the most prevalent non-communicable conditions, affecting people of all ages and often resulting in pain, discomfort, and tooth loss. According to the World Health Organization (WHO), nearly half of the global population is affected by oral health problems. Neglected dental decay in permanent teeth is the most prevalent worldwide condition, affecting roughly 2.3 billion people, followed closely by severe periodontitis, which impacts nearly 1 billion people worldwide (3). Dental caries and periodontitis have a polymicrobial etiology and develop when microbial communities above and below the gumline become imbalanced, leading to dysbiosis and plaque formation. Tooth decay is influenced by two main factors: the immune system and diet. Variations in macronutrient intake and dietary patterns (DPs) can alter the oral microbiota, promoting disease. Nutrients such as sugars, fats, and vitamins play critical roles in shaping the oral microbial composition. In severe cases of early childhood caries, diets high in sugar and frequent snacking are strongly correlated with the presence of *Streptococcus mutans*, a primary cariogenic bacterium (4).

Recent research highlights the association between dietary habits and periodontal disease. Specific DPs and nutrient consumption can activate or modify immune- driven immune responses, which play a role in the onset of periodontal disease. Diets high in carbohydrates and saturated fats exert pro-inflammatory effects and increase the risk of periodontal disease, whereas diets rich in fiber, polyunsaturated fatty acids, antioxidant micronutrients, and calcium exhibit anti-inflammatory properties that may mitigate this risk (Fig. 2) (5). This review aims to examine the interrelationship between oral health, diet, and nutrition.

## Design

### Search strategy and eligibility criteria

This systematic review was conducted in accordance with the applicable elements of the Preferred Reporting Items for Systematic Reviews and Meta-Analyses (PRISMA) guidelines. As a quantitative meta-analysis was not planned, appropriate methodological adaptations were implemented. The search strategy was designed by the authors and executed on December 6, 2024, across the following databases: PubMed, Web of Science (WoS), and Scopus. The search terms included combinations of the descriptors ‘oral microbiota’ or ‘oral microbiome’ with dietary-related terms such as ‘diet’, ‘prebiotics’, and ‘probiotics’, restricting the results to studies conducted in humans and published between 2010 and 2024. The complete search strategy used for each database is presented in Table 1. Original articles were eligible for inclusion if they met the following criteria: 1) conducted in human populations, 2) published in English or Spanish, and 3) assessing the relationship between the oral microbiota and either DPs or supplementation with prebiotics or probiotics. Both observational and interventional study designs were considered. The following were excluded: 1) animal studies, 2) in vitro experiments, 3) reviews, meta-analyses, editorials, commentaries, and conference abstracts, and 4) studies that did not report specific outcomes related to oral microbiota composition or where the dietary exposure or microbiological analysis was not a central objective of the study.

**Table 1:** Search terms used in the different databases

Database	Search terms
PubMed (U.S. National Library of Medicine)	(“oral microbiome”[Title/Abstract] OR “oral microbiota”[Title/Abstract]) AND (“diet”[Title/Abstract] OR “nutritional intervention”[Title/Abstract] OR “prebiotics”[Title/Abstract] OR “probiotics”[Title/Abstract]) AND (humans[MeSH Terms]) AND (“2010”[Date - Publication]: “2024”[Date - Publication])
Web of Science Core Collection (Clarivate Analytics)	TS=(“oral microbiota” OR “oral microbiome”) AND TS=(“diet*” OR “prebiotic*” OR “probiotic*”) AND PY=(2010–2024)
Scopus (Elsevier)	TITLE-ABS-KEY(“oral microbiome” OR “oral microbiota”) AND TITLE-ABS-KEY(“diet*” OR “prebiotic*” OR “probiotic*”) AND PUBYEAR > 2009 AND PUBYEAR < 2025

Search terms were adapted to each database’s syntax. Filters were applied to restrict results to human studies and publications between 2010 and 2024. TS: topic search (web of science); TITLE-ABS-KEY: title, abstract, keyword search (scopus); MeSH = medical subject headings (PubMed).

## Results

### Study selection

**Fig. 1:**
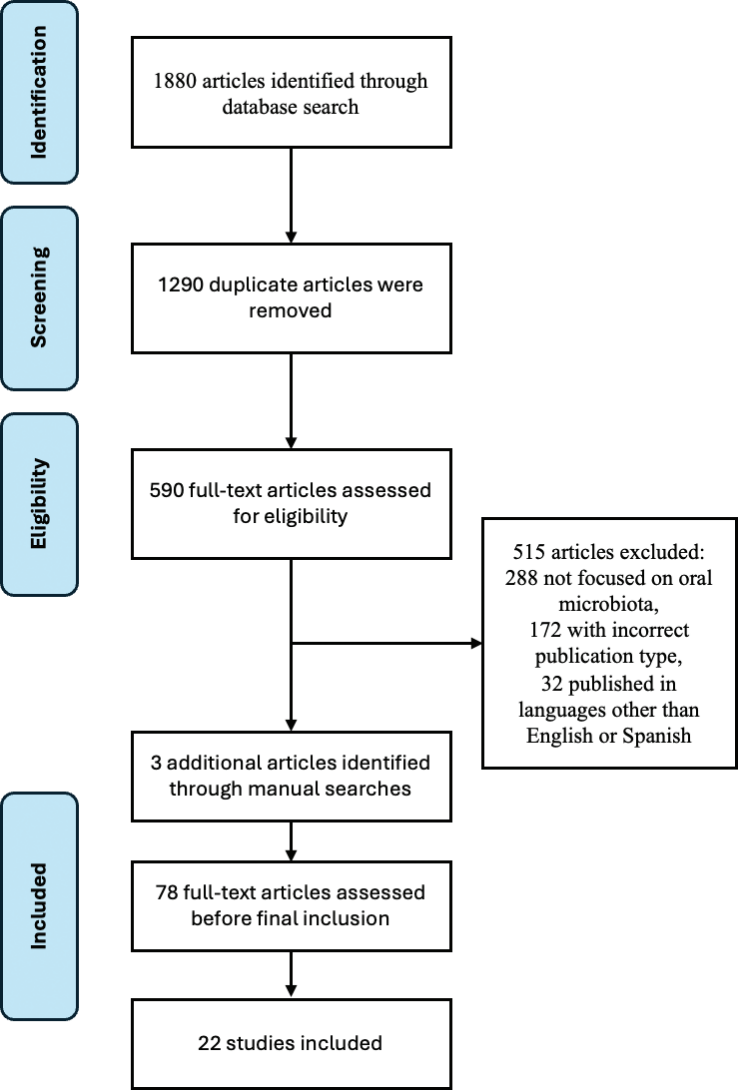
PRISMA flow diagram of the study selection process.

The systematic search initially yielded 1,880 records across three databases (PubMed, Web of Science, and Scopus). After removing 1,290 duplicates, 590 records were screened at full-text level. Of these, 515 were excluded for not meeting eligibility criteria (288 did not focus on oral microbiota, 172 were of incorrect publication types, and 32 were published in languages other than English or Spanish). Seventy-eight articles were assessed in detail, of which 19 were retained. In addition, three articles meeting all eligibility criteria were identified through manual reference checks, resulting in a total of 22 original research articles included in this review. The study selection process is illustrated in the PRISMA flow diagram (Fig. 1).

### Study characteristics

A total of 22 original studies were included in this review. Of these, eight were randomized controlled or pilot clinical trials, four were cohort or longitudinal studies, and 10 were cross- sectional studies. Most were conducted in Europe or North America and published between 2015 and 2024. Sample sizes ranged from 16 to 158 participants, including both healthy individuals and those with conditions such as obesity or periodontal disease. Thirteen studies assessed the effects of specific DPs (e.g. Mediterranean, vegetarian, Western), five evaluated probiotics or synbiotics, and four focused on prebiotic supplementation. A detailed summary of study designs, interventions, and outcomes is presented in Tables 2–4.

**Table 2:** Dietary patterns and effect on oral microbiome

Objective	Study Design	Number and age of participants	Intervention	Parameters evaluated	Outcome	Ref.
To Evaluate the changes in the salivary microbial communities in overweight/obese subjects after an individually tailored MD based nutritional intervention.	Randomized, parallel, assignment trial	49 patients aged 20–60 years.	Subjects were randomly assigned to receive a MD (*n* = 29) or control (*n* = 20) diet for 8 weeks.	Saliva sampling (DNA extraction and 16s rRNA gene sequencing). Dietary habits by weighed food diaries. Clinical parameter (hs-CRP).	Significant decrease of *Porphyromonas gingivalis, Prevotella intermedia*, and *Treponema denticola*. Increased levels of *Streptococcus cristatus*.	(30)
To analyze the hypothesis that reduced inflammation associated with a vegetarian diet would promote a more commensal subgingival bacterial profile.	Cross-sectional trial	39 patients aged > 50 years	Vegetarians: never or rarely consuming meat. Non-vegetarians: consuming non-fish meats 1 time per month or more and all meats combined more than 1 time per week.	Plaque samples (DNA extraction and 16s rRNA gene sequencing).	Increased levels of *Actinobacteria*, and *Proteobacteria* in vegetarian group. Significant increased levels of *Mogibacterium timidum* and *Veillonella rogosae* in non-vegetarians.	(37)
To investigate the effects of an oral health optimized diet on the composition of the supragingival oral plaque.	Randomized controlled pilot trial	16 patients aged 18–75 years	Experimental group changed a high carbohydrates diet to a diet rich in antioxidants and fiber for 4 weeks.	Saliva and supragingival plaque samples	Significant decrease of *Streptococcus mitis, Granulicatella adiacens, Actinomyces* spp. y *Fusobacterium* spp. in plaque samples of the experimental group.	(42)
To assess two macronutrient interventions in a 2x2 factorial dietary design to determine their effects on oral health.	Single-blinded, parallel randomized trial	67 patients aged 65–75 years	Omnivorous (50% animal and 50% plant protein) vs semi-vegetarian (70% plant and 30% animal protein) diet for 4 weeks.	Supra and subgingival plaque (DNA extraction and 16s rRNA gene sequencing).	Beneficial, but no significative effects on bacterial alpha diversity for all diets in supra and subgingival plaque pre and post- intervention.	(44)
To identify salivary microbiota cluster groups and identify lifestyle and host factors which were associated with these groups.	Exploratory study with a clinical sample	176 patients aged 17–21 years	Energy intake: 1856 kcal/day, with 40.3% coming from carbohydrates Sucrose intake: 27 g/day	Saliva Sampling, Bacteria culturing (DNA Extraction and 16s rRNA gene sequencing).	Increased levels of *Actinomyces, Bifidobacterium* and *Veillonella*, and *S. wiggsiae, S. mutans*, and *S. sobrinus* in groups with high sugar intake.	(51)
To understand the association between carbohydrate intake and the oral microbiome with caries and periodontal disease.	Prospective cohort trial	1204 patients aged > 50 years	Food frequency questionnaire during 3 months.	Subgingival plaque samples (DNA extraction and 16s rRNA gene sequencing). Bacterial alpha diversity.	Positive association between sucrose with *S. mutans* and *Streptococcus lactarius*.	(52)
To investigate the effects of sucrose, milk, yoghurt and a diet rich in dietary fiber on the oral biofilm microbiota.	Randomized controlled trial	11 patients aged 21–56 years	Food frequency questionnaire during 7 weeks.	Supragingival plaque sample (DNA extraction and 16s rRNA gene sequencing).	Increased levels of *Streptococcus mitis* and *Streptococcus infantis* in group with high sugar intake	(53)
To assess the composition of the oral microbial flora of adults with rampant caries.	Case-control trial	60 patients aged 16–70 years	Association between a high sugar diet and patients with rampant caries.	Supragingival plaque sample (DNA extraction and 16s rRNA gene sequencing).	Increased levels of *Veillonella* and *Leptotrichia* in group with high sugar intake	(80)
To determine the effect of chewing gum containing xylitol and freeze-dried blackberry powder on oral bacteria	Randomized, controlled, cross-over trial	50 patients aged > 18 years	Intervention: 700 mg xylitol and 50 mg freeze-dried	Saliva Sampling. Quantitative polymerase chain reaction (qPCR).	Significant decrease of *F. nucleatum, P. intermedia* and *L. Buccalis* in saliva samples of the group with xylitol intake.	(56)
To test the hypothesis that the short-term use of xylitol-containing chewing gum reduces total salivary bacteria count.	Randomized controlled trial	70 patients aged ≥ 20 years	Intervention: xylitol 7 g/day for 2 days	Saliva Sampling. (DNA extraction and 16s rRNA gene sequencing).	Significant differences between the baseline and follow-up samples (*Prevotella* and *Neisseria*)	(81)

MD: mediterranean diet.

**Table 3:** Probiotics on oral health

Objective	Study design	Number and age of participants	Intervention	Parameters evaluated	Outcome	Ref.
Evaluate the benefits of *Lactobacillus rhamnosus* and *Bifidobacterium lactis* on gingivitis	Four-week, randomized double blind, placebo-controlled trial	108 patients aged 13-15 years	Supplementation with pills composed of 4.8 x 108 CFU twice a day	Gingival index, index plaque and determination of the periodontal pathogens A. *actinomycetemcomitans, P. gingivalis, P. intermedia*, and *F. nucleatum*	Significative reduction in gingival index, and periodontal pathogens	(68)
To investigate the effects of *Lactobacillus reuteri* on oral health	Forty-five days, randomized double blind, placebo-controlled trial	27 patients aged 12-18 years	Daily supplementation with tablets containing 1 x 108 CFU	Salivary pH, *Streptococcus mutants* and *Lactobacillus* levels, plaque accumulation, gingival index and bleeding on probing	Beneficial, but no significative effects on levels of *Lactobacillus sp*.	(69)
Efficacy of *Bifidobacterium animalis* against gingivitis	Eight-week, randomized double blind, placebo-controlled trial	60 patients aged > 20 years	Experimental group take 1 x 109 CFU tablets daily twice a day	Bleeding on probing, probing pocket depth, index plaque, gingival index and cytokine levels IL-1α, IL-1β, MCP-1, MIP-1β and IL-8	Significative reduction on bleeding on probing, gingival index, IL-1α, and, MCP-1	(70)
To evaluate the clinical efficacy of *Streptococcus dentisani* to promote oral health	Four-week, randomized double-blind, placebo-controlled clinical trial	59 patients aged 18-65 years	Application of bucco-adhesive gel every 48 hr containing 2.5 x 109 CFU	Plate index, gingival index, salivary flow and S. dentisani levels	Beneficial shift in bacterial composition with reduction of cariogenic microorganisms and significative increase of salivary flow, calcium and ammonium associated with oral health	(67)
To assess the efficacy of *Lactobacillus salivarius* administration on halitosis	Four-week, randomized pilot study	20 patients aged 30-66 years	Daily administration of 3 tablets equivalent to 2 x 109 CFU	Bleeding on probing, probing pocket depth, index plaque, halitosis by subjective test and gas chromatography, blood and microbiology of saliva	Significant improvement of physiologic halitosis and beneficial effects on bleeding on probing from the periodontal pocket. *Lactobacillus salivarus* increases and blood diminished in saliva.	(64)
To identify the effects of *Weisellia cibaria* tablet ingestion on halitosis and psychosocial factors	Eight-week, randomized placebo-controlled trial	62 patients aged >20 years	Experimental group take a tablet of 1 x 108 CFU daily before bed	Subjective measurements related with oral health, depression, self-esteem and quality of life	Significative reduction in halitosis and improvement in quality of life related with oral health	(65)
To evaluate the efficacy of *Lactobacillus salivarius, Lactobacillus paracasei* and *Lactobacillus plantarum* to improve oral health and immunity	Four-week, randomized double blind, placebo-controlled trial	50 patients aged 20-40 years	Daily ingestion of pills containing 3 x 109 CFU	Plaque accumulation, oral health questionary, IgA, pathogen and commensal levels	Significative increment of IgA and *Lactobacillus* in buccal cavity. Improvement in oral and intestinal health and reduction in plaque accumulation	(74)
To determine the potential protection of *Lactobacillus rhamnosus* and *Lactobacillus curvatus* against experimental gingivitis	Four-week, randomized double blind, placebo-controlled trial	80 patients aged 19-33 years	Daily supplementation with tablets containing 1 x 109 CFU	Oral microbiota, gingivitis, bleeding on probing, index plaque, proteases and cytokines	Probiotics reduce the resolution of experimental gingivitis improving microbiota composition	(72)
Determine *Lactobacillus plantarum* impact on gingivitis therapy	Four-week, randomized triple blind, placebo-controlled trial	42 patients aged >18 years	Probiotic mouthwash consisting of 20 ml containing 1 x 108 CFU	Plaque index, gingival index, bleeding on probing and oral hygiene index	Gingival index showed a significant difference in the fourth week	(71)
To evaluate if *Lactobacillus rhamnosus* affects the levels of oral microbiota and colonize the human mouth	Four-week, randomized controlled clinical trial	43 patients aged 20-25 years	Daily consumption of fermented milk containing 1 x 106 CFU	An oral examination was performed and the numbers of mutans *streptococci, lactobacilli*, and total bacteria in saliva were counted. The persistence of *L. rhamnosus* was investigated using PCR	Significative reduction on total bacteria and *S. mutans* concentration. Increase of *Lactobacilli* and persistence of *L. rhamnosus* suggesting beneficial effects on oral health	(75)
To assess if *Lactobacillus reuteri* prevents periodontal deterioration	Forty-two days, randomized double blind, placebo-controlled trial	204 subjects aged 18-65 years	Supplementation with pills composed of 1 x 108 CFU twice a day	Bleeding on probing, gingival index, plaque control record, probing attachment level, and probing pocket depth	The test group significantly improved when compared to baseline in all assessed parameters	(73)
To investigate the effect of *Bifidobacterium lactis* on dental caries	Two-week, randomized double-blind, placebo-controlled clinical trial	66 patients aged 18-30 years	Daily consumption of yogurt containing 1 x 106 CFU	Levels of *Streptococcus mutans* and *Lactobacilli* in saliva	A significant reduction in salivary *S. mutans* counts was observed suggesting a positive modification of the oral biofilm	(66)

CFU: colony-forming unit; IL-1α: interleukin-1 alpha; IL-1β: interleukin-1 beta; MCP-1: monocyte chemoattractant protein-1; MIP-1β: macrophage inflammatory protein-1 beta; IL-8: interleukin-8.

**Table 4:** Reported oral prebiotics

Identified prebiotic	Food source	Inhibited microorganism
Arginine	Nuts, chickpea, lentils.	*Candida. S. mutans*
Polyalcohols	Fruits and vegetables, fermented products	*Xylitol reduces the level of S. mutans*
Nitrates	Fruits, vegetables, sausages	*S. mutans, Casei, A. naeskundii, also F. nucleatum, Eikenella corrodens and Porhyromonas gingivales*
Xilooligosaccharides, Galactooligosaccharides, Fructooligosaccharides.	Breast milk, fruits and vegetables	*S. mutans*
Lactose	Milk, yogurt	S. mutans
Inulin	Asparagus, bananas and leeks	*NR*
D-tagatose (non cariogenic sugar)	Chewing gum	*S. mutans, S. gordonii*
Arginine	Exists in saliva as free acid or as peptide constituents	*Candida*

### Oral microbiome, eubiosis and diet

The human oral cavity serves as a primary ecological niche for diverse microbial communities that interact with both the external environment and internal physiological systems. The oral microbial community represents a crucial component of the human microbiome (6). The oral microbiome, recognized as the second largest and most varied microbial ecosystem after the gastrointestinal tract, refers to the community of microorganisms residing in the mouth (7). It is among the body’s most stable and complex ecosystems, supported by the diversity of niches within the oral cavity and a consistent influx of external nutrients from food intake and internal nutrients from saliva production (8). It accounts for approximately 700 species of viruses, protozoa, fungi, bacteria, and archaea (9), colonizing various surfaces within the oral cavity such as teeth, tongue, inner cheeks, tonsils, gingival sulcus, and both the hard and soft palates (10). Colonization of the intestines is initially seeded by microbial populations originating from the oral cavity.

Because the mouth is continuously exposed to the external environment, it harbors diverse microorganisms that form a complex community, playing an essential role in both dental health and disease processes affecting the teeth and gums (6, 10). The oral microbiota engages in bidirectional communication with the host, indicating immunological and metabolic health across the mouth and other bodily organs. Various external factors, such as dietary habits, can modulate microbiome composition, underscoring the significant role of a balanced diet in supporting the oral microbiota’s eubiosis (11). Oral health is governed by the equilibrium between beneficial (commensal) and harmful (pathogenic) bacteria within the oral cavity. In this context, the commensal microbiota predominates (12). The primary components of the oral microbiota are bacteria. A mere 1 mL of saliva contains about 108 microbial cells, representing around 100–200 bacterial species in a healthy oral microbiome. Core bacterial genera, which are present in over 66% of healthy human mouths, include *Haemophilus, Neisseria, Granulicatella*, and *Streptococcus*. Dominant bacterial groups in healthy oral microbiomes include *Bacteroidetes, Firmicutes, Spirochaetes, Fusobacteria, Actinobacteria*, and *Proteobacteria* (13). Primary colonizers are mainly anaerobic, Gram-positive oral *Streptococci*, such as *Streptococcus mitis, Streptococcus oralis*, and *Streptococcus sanguinis*, followed by Gram-positive rod-shaped species like *Actinomyces*. The oral cavity relies on a continuous balance between commensal microbial populations to sustain a healthy oral environment (14). Diet supplies essential nutrients for the oral microbiota and influences the survival and proliferation of specific microbial communities, promoting the maintenance and reproduction of microorganisms that are most capable of utilizing food resources obtained from the host (15). Given that changes in the host’s lifestyle can lead to alterations in the microbiome, it is crucial to understand the potential effects that may arise from the introduction of new dietary factors, environmental stimuli, or novel pharmaceutical treatments. Bacteria that contribute to optimal oral health, such as *Streptococcus mitis*, thrive in environments with low sugar concentrations. These microorganisms, operating within diverse microbial communities, are capable of colonizing oral surfaces and preventing pathogenic microorganisms from adhering to and proliferating on these surfaces (13). More specifically related to diet, the physical and chemical characteristics of the foods we consume, as well as factors like their consumption patterns (e.g. frequency, method of intake), can either support or negatively affect oral health, particularly the teeth and supporting structures. For example, sugar alcohols (e.g. xylitol), hard cheese, whole grains, fruits, and high-quality proteins, along with sufficient intervals between meals, may offer protection against dental caries (16). Dairy products like yogurt, cheese, and milk are generally considered to have no cariogenic potential, likely due to their high phosphate and calcium content, buffering capacity, casein phosphopeptides, lactose, and casein. Additionally, it is suggested that lipids from dairy may protect enamel by forming a coating that prevents tooth enamel demineralization (17). A diet rich in fruits, fiber, polyunsaturated fatty acids, antioxidant micronutrients, vegetables, and calcium exhibits anti-inflammatory effects and may help reduce the risk of periodontal disease (Fig. 3) (5).

**Fig. 2:**
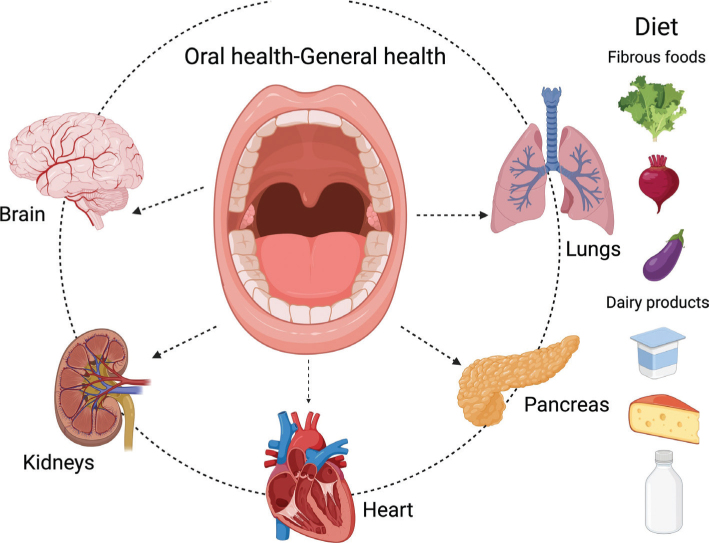
Oral health, general health, and diet.

**Fig. 3:**
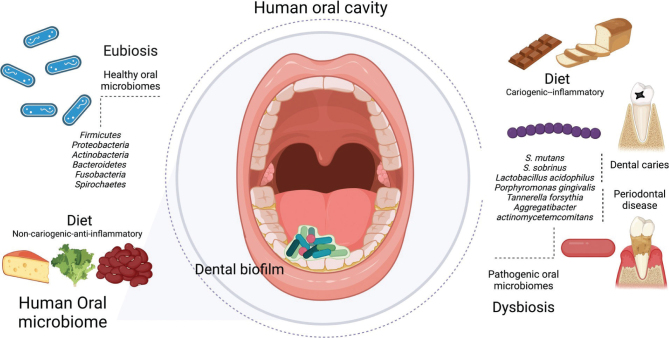
Human oral microbiome, eubiosis, and dysbiosis diet.

### Oral microbiome, dysbiosis, and diet

When oral homeostasis is disturbed and beneficial bacteria no longer predominate, the environment becomes conducive to dysbiosis, promoting the proliferation of bacterial species, with pathogenic traits. Evidence suggests that dysbiosis may precede the clinical manifestation of oral diseases (10). Dental caries and periodontitis have a multifactorial origin and arise when the microbial communities within supragingival and subgingival niches lose their balance, leading to dysbiosis. This imbalance promotes the formation of a biofilm (plaque) on the teeth and surrounding tissues. The resulting biofilm serves as a protective matrix, shielding pathogenic microorganisms from the host’s immune defenses and contributing to the development of dental infections (14). The pathogenic bacteria most associated with dental caries include Gram-positive *mutans streptococci* (*S. downei, S. rattus, S. mutans, Streptococcus cricetus* and *Streptococcus sobrinus*) as well as lactobacilli present in supragingival biofilms. The primary bacterial strains implicated in caries are *Lactobacillus acidophilus* and *S. mutans*, with the former likely initiating tooth decay and the latter contributing to its progression (4). Increased intake of fermentable carbohydrates, notably free sugars present in processed foods and sweetened beverages, is strongly linked to a higher risk of dental caries. Several keystone pathogens, including *Aggregatibacter actinomycetemcomitans, S. mutans, Tannerella forsythia*, and *Porphyromonas gingivalis*, have been implicated in the etiology and progression of periodontal disease. A DP dominated by foods such as meats from farmed animals, sugar-sweetened dairy products, purified plant oils, and ultra-processed refined grains has been linked to detrimental shifts in the oral microbiota. These alterations, which include an increase in acidogenic and acid-tolerant bacteria, are associated with a heightened risk of periodontal disease (15).

### Dietary patterns and oral health

**Fig. 4:**
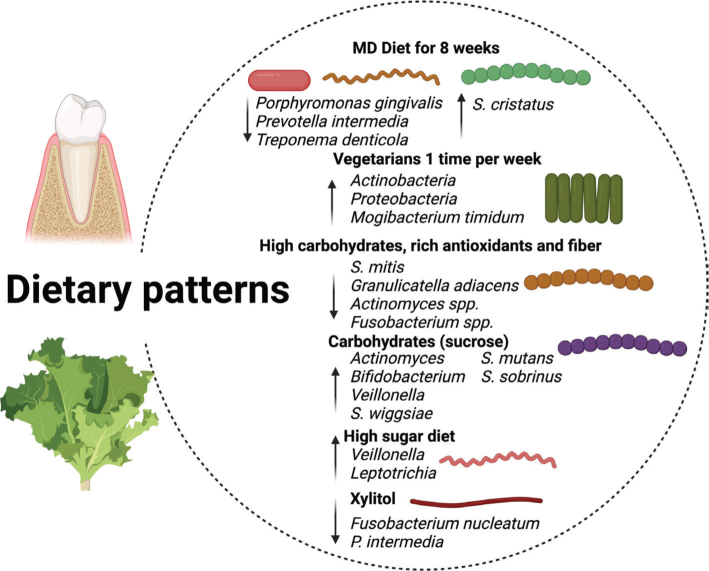
Dietary patterns and oral health.

Traditionally, studies on the relationship between diet and health have emphasized the associations between health outcomes and specific foods or food groups. However, since individuals consume foods and beverages in combination, epidemiological research has increasingly shifted its focus toward DPs (18). A DP refers to the overall composition of the diet, including the types and proportions of food groups, nutrients, and individual foods, as well as the frequency and quantity of their consumption (19). The complex interplay between oral health and dietary habits is fundamental to overall health and quality of life. Oral health is both a marker and a determinant of general health outcomes and is closely tied to nutritional behavior (Fig. 4) (20). Dietary practices significantly influence the health of the oral cavity (21). The impact of dietary transitions on the oral microbiome has been studied by examining bacterial shifts associated with the evolution from hunter-gatherer diets to carbohydrate-rich agricultural diets (22). In contemporary settings, Western DPs, characterized by high sugar consumption, have been associated with deteriorated oral health due to shifts in the relative abundance of oral bacterial populations (Table 2) (23).

### Mediterranean diet

The diversity of foods and DPs can influence the composition of the oral microbiota. In this context, DPs such as the Mediterranean diet (MD) have been evaluated. The MD is characterized by a traditional eating pattern rich in plant-based foods, seafood, and olive oil, typical of the Mediterranean region. It emphasizes a low intake of red meat and highly processed foods. Elevated polyphenol intake through olive oil may enhance its health-promoting effects (24). The MD pattern also includes moderate amounts of eggs and dairy, low to moderate servings of seafood and poultry, minimal red meat, and moderate wine consumption, typically with meals (7). Most studies have focused on identifying the composition and microbial diversity of the oral microbiota, while relatively few have investigated the influence of host genetics and environmental factors, including DPs, such as the MD, on its biological functioning. To date, the effects of the MD have been studied primarily in the gut microbiome. For example, a diet resembling the Mediterranean pattern has been associated with a greater abundance of fiber-degrading bacteria, such as *Faecalibacterium sp*. and *Ruminococcus sp*., along with lower levels of fecal calprotectin in asymptomatic first- degree relatives of patients with Crohn’s disease (25). The oral microbiota is the second most complex microbial ecosystem in the human body after the gut (26). Its complexity arises from the wide range of microorganisms present, including bacteria, fungi, and viruses (27). More than 600 microbial species inhabit distinct ecological niches within the oral cavity, of which 169 comprise the native core oral microbiome. These species are distributed across three main niches: buccal mucosa, including saliva, and supragingival plaque (28). Moreover, the composition of the oral microbiota varies among different oral sites, making it challenging to define a single healthy microbiota profile (29). Individual variation in the oral microbiota is influenced by genetic and environmental factors, including diet. In this regard, Laiola et al. examined salivary microbiota changes in overweight and obese individuals following a personalized MD-based dietary intervention. A reduction in the relative abundance of species-level operational taxonomic units, including Treponema denticola, Prevotella intermedia, and Porphyromonas gingivalis, was observed in the group that followed the MD, compared to individuals who did not change their lifestyle. These bacteria are commonly associated with periodontal disease. Additionally, increased levels of Streptococcus cristatus were found in the MD group, a microorganism known to antagonize P. gingivalis by downregulating its virulence gene expression (30). Despite the well-documented benefits of the MD, adherence to this DP appears to be declining in Western countries (31). In recent years, a notable reduction in adherence has been reported among the Spanish population, accompanied by a growing interest in alternative dietary models such as vegetarian or plant-based diets (32).

### Vegetarian diet

The vegetarian diet is another DP that has been recognized for its positive effects on various health outcomes, including a reduced risk of chronic diseases (33). This pattern is typically defined by the exclusion of certain animal-derived foods, such as red and processed meats, and a higher intake of plant-based foods (34). Vegetarian diets are associated with more favorable scores on the Dietary Inflammatory Index (35), reflecting a reduced pro-inflammatory potential (36). Additionally, shifts in the gut microbiota may influence the anti-inflammatory properties attributed to vegetarian diets. It has been proposed that the anti-inflammatory effects of a vegetarian diet may also impact the composition of the subgingival microbiota. In a study by Khocht et al. vegetarian individuals exhibited a positive correlation between the anti-inflammatory cytokine interleukin-10 and two bacterial species associated with periodontal health, Rothia aeria and Peptidiphaga sp. HMT183 (37). The Peptiphaga genus is commonly found in subgingival biofilms linked to periodontal stability, while Rothia species have been associated with both periodontal health and certain systemic infections, including prosthetic and native endocarditis (38, 39). Compared to non-vegetarians, vegetarians tend to consume more antioxidants and dietary fiber (40). The relationship between fiber intake and oral health has mostly been addressed from a correlational perspective. For instance, a study evaluating fiber intake in adults reported that the consumption of whole grains was significantly associated with a reduced prevalence of periodontal disease (41). In an intervention study, Tennert et al. demonstrated that a high-fiber diet sustained over 4 weeks led to a reduction in specific bacterial taxa in supragingival plaque, including *Granulicatella adiacens, S. mitis, Fusobacterium spp*., and *Actinomyces spp*. Previous studies have indicated that *S. mitis* and *G. adiacens* may contribute to periodontal disease development (42). However, findings on the broader impact of vegetarian patterns are not entirely consistent (43). For example, Eberhard et al. found no significant changes in bacterial alpha diversity following a 4-week intervention with a semi-vegetarian diet composed of 70% plant-based and 30% animal protein sources (44).

### High sugar diet

High sugar intake has been identified as a key dietary factor contributing to the development of dental caries by altering the microbial composition of dental plaque (45). The frequent consumption of sugar-rich beverages, which are often acidic and high in fermentable carbohydrates, fosters a favorable environment for the proliferation of acidogenic bacteria through fermentation processes. These conditions promote the dominance of microbial species that thrive in acidic environments, facilitating enamel demineralization and disease progression (46, 47). Recent research has described a high- sugar DP characterized by the differential intake of various sugar types, including fructose, glucose, sucrose, maltose, and lactose (48). Among these, sucrose plays a particularly critical role in disrupting microbial homeostasis within oral biofilms (49). It promotes the growth of acid-producing bacteria while suppressing the presence of alkali- producing species, thus shifting the community structure towards cariogenic profiles. This imbalance leads to increased colonization by acid-resistant bacteria (50). For example, Esberg et al. reported that elevated sucrose intake was associated with an increased abundance of *S. sobrinus, Bifidobacterium, S. wiggsiae, Veillonella, S. mutans*, and *Actinomyces*, all of which are known contributors to caries development in adults (51). Similarly, Millen et al. found a positive correlation between sucrose consumption and the prevalence of *S. mutans* and *Streptococcus lactarius* (52). Moreover, *Streptococcus infantis* has been implicated in caries development, with evidence showing that an increase of at least 10 g of sugar per day over 7 weeks significantly elevates its abundance (53). Although sugar-rich diets are known to reduce microbial diversity, some bacteria such as *Veillonella* require additional compounds like lactate to grow. While *Veillonella* has previously been considered protective against dental caries due to its metabolic role (54, 55), emerging evidence suggests that it may also contribute to adverse oral health outcomes (56). Considering these findings, adopting a healthy lifestyle that includes a balanced diet supportive of microbiota regulation is essential for maintaining oral health. Natural sweeteners such as xylitol have gained attention as potential alternatives. Xylitol has been shown to inhibit the growth of pathogenic bacteria including *F. nucleatum, P. intermedia*, and *Prevotella* (57, 58). Chewing gum containing xylitol has received interest due to its practical application. Short-term use may help reduce the secretion of pro-inflammatory cytokines and suppress the presence of *S. mutans* (59). Long-term use has also been associated with sustained reductions in *S. mutans* levels and cytokine expression, supporting its role as an effective adjunct in oral health maintenance (60, 61). Although the Mediterranean and Western diets were the most frequently assessed DPs, one study also evaluated a vegetarian diet. No eligible studies were identified which explored the effects of other recognized DPs (e.g. DASH, ketogenic, low-carbohydrate) on oral microbiota. This absence may reflect limited research or the exclusion of such studies due to eligibility criteria.

### Probiotics and prebiotics in an oral health

#### Probiotics

The term probiotic was introduced in 1965 by Lilly and Stillwell to describe a substance secreted by one organism which supports the development of another (62). Metchnikoff later hypothesized that the extended lifespan of the Bulgarian population was linked to the consumption of fermented foods containing lactic acid bacteria, which contributed to enhanced gastrointestinal health (63). The current definition of probiotics aligns with Metchnikoff’s concept, describing them as viable microorganisms that, when ingested in adequate amounts, confer health benefits to the host (64). Numerous investigations have emphasized the role of specific probiotic strains in supporting gastrointestinal, genitourinary, and oral health by maintaining ecological balance within these environments (65). The oral mucosa represents a dynamic microbial habitat that relies on ecological equilibrium to preserve health. When factors such as poor oral hygiene or dietary imbalances disrupt this homeostasis, oral diseases may emerge. Therefore, the introduction of beneficial bacterial strains into the oral cavity is considered a promising strategy to restore microbial balance and prevent or manage oral conditions (65). The therapeutic potential of probiotics in oral health encompasses halitosis, gingivitis, caries, periodontitis, mucosal immunity, and dysbiosis. Most studies have focused on the effects of *Lactobacillus* and *Bifidobacterium* strains; however, some investigations have also explored formulations containing *Streptococcus* or *Weisellia*. The most common delivery systems include capsules, tablets, pills, fermented milk, yogurt, and, less frequently, gels or solutions. Clinical trials in adults have generally reported improvements in oral health, assessing a range of parameters (Table 3). For example, 4 weeks of supplementation with *Lactobacillus salivarius* effectively reduced halitosis, as measured by subjective assessments and gas chromatography, and also showed benefits in bleeding during periodontal probing and salivary blood concentration (66). Similarly, tablets containing *Weisellia cibaria* improved halitosis and contributed to better oral health, thereby enhancing quality of life after 8 weeks (67). In a randomized, double-blind, placebo-controlled trial, the daily intake of 300 g of yogurt enriched with *Bifidobacterium lactis* Bb12 for 2 weeks resulted in a significant reduction in salivary *S. mutans* and *Lactobacillus counts* among young adults with initial stages of dental caries. Compared to baseline and the control group (who consumed conventional yogurt), the intervention group demonstrated measurable improvements in oral microbiota composition, suggesting a favorable modulation of the oral biofilm (68). Likewise, a 1-month intervention with *Streptococcus dentisani* (2.5 × 10^9^ CFU per dose, applied every 48 h) demonstrated a reduction in cariogenic microorganisms along with an increase in salivary flow, calcium, and ammonium ions, which are factors associated with enhanced oral health (69).

Regarding gingivitis, supplementation for 1 month with *Lactobacillus rhamnosus* and *Bifidobacterium lactis* reduced the gingival index, plaque index, and levels of periodontal pathogens (70). In another study, *Lactobacillus reuteri* administered for 45 days (strains DSM 17938 and ATCC PTA 5289, minimum 5 × 10^8^ CFU each) improved plaque accumulation and gingival bleeding, and lowered *S. mutans* levels, although the latter change did not reach statistical significance (71). Conversely, *Bifidobacterium animalis* supplementation at a dose of 1 × 10^9^ CFU/day for 2 months significantly reduced bleeding observed during probing, gingival inflammation, and levels of pro-inflammatory cytokines such as IL-1α and MCP-1 in adults with gingivitis. These effects were observed after twice-daily ingestion of tablets, highlighting its potential as a therapeutic adjunct in the management of periodontal inflammation (72). Additional benefits have been observed with *Lactobacillus plantarum*, which lowered the gingival index after 1 month (73), and with *L. rhamnosus* (10^9^ CFU/day each) for 12 weeks) and *Lactobacillus curvatus*, which shortened the duration of experimentally induced gingivitis and improved microbiota composition (74). A 42-day supplementation with *L. reuteri* also prevented periodontal deterioration, as evidenced by improvements in bleeding on probing, plaque scores, attachment levels, and probing pocket depth (75). A combined probiotic formulation containing *L. salivarius, Lactobacillus paracasei*, and *Lactobacillus plantarum* not only reduced plaque accumulation but also enhanced IgA production and increased salivary *Lactobacillus levels*, suggesting immunomodulatory effects alongside microbiota optimization (76). In a double-blind, randomized, placebo-controlled trial, supplementation with 100 mL/day of milk fermented with *L. rhamnosus* GG (10^9^ CFU/mL) for 7 months significantly reduced the number of plaque *S. mutans* in preschool children.

Compared to baseline, the intervention group showed a reduction in plaque *S. mutans* by nearly 50%, indicating a potential protective effect against dental caries (77). Overall, the majority of studies report beneficial effects of probiotics on oral health, with few or no adverse events. Nevertheless, further research is required to determine the optimal strains, dosages, and intervention durations necessary to achieve specific therapeutic outcomes.

#### Prebiotics

The type and frequency of food and beverage consumption directly influence oral pH and microbial dynamics, thereby contributing to the development of dental caries (78). A prebiotic is defined as a non-digestible food component that promotes microbial balance by selectively stimulating the growth or activity of beneficial microorganisms in the oral cavity, ultimately conferring a health benefit. Evidence indicates that prebiotics may enhance the effectiveness of probiotics in the management of oral diseases (79). This synergistic interaction offers several advantages, as the prebiotic acts as a selective substrate that promotes the proliferation of specific probiotic strains, depending on both dosage and microbial species (Table 4) (79, 80). To be classified as a prebiotic, a dietary component must meet the following criteria: 1) resistance to hydrolysis and absorption in the upper gastrointestinal tract, 2) stability against gastric acidity and digestive enzymes, and 3) the ability to exert a physiologically beneficial effect on host health (81). Adopting healthy DPs, particularly those rich in dietary fiber, may contribute to the prevention of periodontal disease. In this regard, Nielsen et al. reported that sufficient intake of fruits and vegetables is associated with a lower incidence of periodontal disease among American adults aged 30 years and older (41).

While dietary components and microbial supplementation strategies play a crucial role in modulating the oral microbiome, everyday behavioral practices such as oral hygiene routines also exert a significant influence on the composition and stability of oral microbial communities:

#### Effects of toothpaste and antimicrobial mouthwashes on the human oral microbiome

A well-balanced oral microbiome and curbing the growth of dental plaque is linked to good oral health, while disturbances can lead to imbalances connected to oral diseases. Thus, it is crucial to comprehend how the regular use of oral hygiene products affects the microbiome (82). In this context, one of the most efficient methods to prevent oral diseases is the treatment of biofilm means of effective oral hygiene practices, typically achieved through mechanical methods such as toothbrushing, flossing, and interdental brushing. Mechanical approaches can be supported with the use of antiseptic formulations in toothpastes or mouthrinses (83). To illustrate this, Hong et al. evaluated through a randomized clinical trial dental calculus inhibition with pyrophosphate toothpaste and its effect on oral microbiome changes. Eighty subjects were allocated to the test group to which pyrophosphate-containing toothpaste was given or the placebo control group. Plaque index, gingival index, calculus index, probing depth, and bleeding on probing were measured at the baseline, and at 4, 8, and 12 weeks. Genomic DNA was extracted from the plaque samples collected at the baseline and at 12 weeks, and 16S ribosomal RNA gene amplicon sequencing was applied for microbiome analysis. A significant difference of microbiome between the baseline and 12 weeks was observed in the test group. Between baseline and 12 weeks, the proportion of *Spirochetes* decreased in the control group, and the proportions of *Proteobacteria, Fusobacteria*, and *Spirochetes* in the phylum level and the proportions of *Haemophilus, Fusobacterium*, and *Capnocytophaga* in the genus level decreased in the test group. In the test group, as plaque index decreased, *Streptococcus* increased, and *Fusobacterium* and *Haemophilus* parainfluenza decreased. The pyrophosphate-containing toothpaste effectively inhibited the dysbiosis of the oral microbiome and the proliferation of pathogenic species in periodontal disease. They concluded that the pyrophosphate-containing toothpaste effectively inhibited oral microbiome dysbiosis and the proliferation of pathogenic species in periodontal disease. They concluded that the pyrophosphate-containing toothpaste effectively inhibited oral microbiome dysbiosis and the proliferation of pathogenic species in periodontal disease. Clinically, plaque formation in the pyrophosphate-containing toothpaste group was effectively reduced (84). Similarly, Kong et al. conducted a study to evaluate the influence of theaflavins (TF), the main bioactive component of black tea, on the diversity of the oral microbiota. In this study, 80 samples collected from the saliva and supragingival plaque of 20 healthy adults using toothpaste with the absence or presence of TFs for a period of 4 weeks were used for the oral microbiome analysis by 16S rRNA gene sequencing. Alpha and beta diversity analysis showed that tooth brushing using the toothpaste with TFs significantly increased the microbial abundance in the saliva samples, and altered the oral microbiota obtained from the saliva and supragingival plaque. The linear discriminant analysis revealed that the use of toothpaste with TFs significantly reduced the abundance of oral pathogens (e.g. *Prevotella, Selenomonas*, and *Atopobium*) while it increased the abundance of oral-health associated bacteria (e.g. *Streptococcus* and *Rothia*). In addition, using toothpaste with TFs reduced the functional pathways abundance relevance to the extracellular polymeric substance (EPS) synthesis while it enriched the functions in transporters, ABC transporters, two-component system, and amino acid metabolism. Collectively, the results demonstrated evidence for the application of toothpaste containing TFs as a promising oral care product (83). Likewise, Wiatrak et al. evaluated the effect of toothpaste containing natural tea tree essential oil (TTO) and ethanolic extract of propolis (EEP), on microflora and selected indicators of oral health in patients using removable acrylic partial dentures. Fifty patients with varying conditions of hygiene were divided into two groups. The study group received the toothpaste with TTO and EEP, while the control group received the same toothpaste but without TTO and EEP. The number of isolated strains of microorganisms in the study group was decreased or maintained at the same level, whereas in the control group an increase in the number of isolated strains was observed. The observed stabilization of oral microbiota in patients from the study group confirmed the beneficial activity of toothpaste containing EEP and TTO compared to the control group (85).

In another investigation, Adams et al. analyzed the composition and activity of dental plaque microbiome from 115 participants after brushing with two toothpastes, one containing zinc citrate trihydrate and the other a control toothpaste, in a parallel design. Analysis of microbiome function based on metagenomic and metatranscriptomic analysis show that use of the zinc toothpaste is associated with an in vivo reduction in glycolysis, consistent with the mode of action of zinc and, increases in processes linked to gum-health (lysine biosynthesis), and to whole-body health (nitrate reduction). These findings provide the first understanding of the beneficial modulation of microbiome composition and function by zinc-containing toothpaste in vivo for oral care benefits (82). Furthermore, Iniesta et al. evaluated the microbiological safety and subgingival impact of a toothpaste with cetylpyridinium chloride (CPC) and cymenol, compared to a sodium monofluorophosphate based toothpaste, and assessed overall subgingival microbiome changes after 6 weeks of routine toothbrushing in patients with gingival inflammation. It was concluded that the daily use of a CPC/cymenol toothpaste was microbiologically safe, with no negative effects on the composition of the subgingival microbiome in patients with gingival inflammation, when compared to a fluoride-based toothpaste. The overall composition of the subgingival microbiome was not significantly affected by the daily use of either toothpaste after 6 weeks (86).

Finally, Zhang et al. evaluated the short-term impact of fluoride toothpaste with different fluoride concentrations (standard fluoride 1,000 ppm, low fluoride 500 ppm, and non-fluoride) on the oral microbial community in preschool children. A total of 48 children participated, with dental plaque samples collected at baseline, and at 1 and 4-week follow-up after using the assigned toothpaste. The microbial community was analyzed using 16S rDNA sequencing to evaluate diversity, composition, and shifts in bacterial populations. The results of the diversity analysis revealed significant differences in microbial composition between the fluoride and non-fluoride groups. Notably, the standard fluoride group exhibited a more substantial shift in bacterial structure, with an increase in the abundance of beneficial species such as *Streptococcus parasanguinis* and *Veillonella*, and a reduction in genus, such as *Haemophilus* and *Neisseria*, which are associated with biofilm formation and may affect the colonization of cariogenic bacteria. These findings suggest that fluoride-containing toothpastes, especially standard fluoride (1,000 ppm) toothpastes, can modulate the oral microbiome by decreasing harmful bacteria and promoting a more balanced microbial environment, potentially reducing the risk of dental caries in preschool children (87).

In addition to toothpaste formulations, currently, there is a wide variety of chemical and natural antiseptic mouthwashes available to help control bad breath (halitosis), tooth decay, and gum disease, which are often used daily. These include chlorhexidine (CHX), hydrogen peroxide (H2O2), CPC, povidone-iodine (PVP-I), and essential oils (EO). A common mechanism by which many antimicrobial mouthwashes eliminate pathogenic bacteria is the destruction of the microbial cell wall, leading to cell death. That commonly used mouthwashes can reduce plaque and gingivitis, with CHX possibly being the most effective (88).

## Discussion

This systematic review analyzed 22 original studies assessing the relationship between dietary factors and the composition of the oral microbiota in human subjects. The findings indicate a growing scientific interest in this topic, particularly over the past decade, with most studies conducted in Europe and North America. While heterogeneous in methodology, the studies collectively suggest that diet plays a key role in modulating the oral microbiome, with potential implications for oral and systemic health. Among the included studies, 12 focused on overall DPs. The MD was the most frequently evaluated, consistently associated with greater microbial diversity and higher abundance of beneficial genera such as *Streptococcus, Veillonella*, and Neisseria. Conversely, adherence to Western DPs, characterized by high intakes of saturated fats, refined carbohydrates, and added sugars was linked to dysbiotic profiles, with increased prevalence of *S. mutans* and other acidogenic bacteria associated with caries and periodontal disease. Vegetarian and plant-based diets also showed potential benefits, though the number of studies remains limited. Only one study directly evaluated a vegan diet, suggesting a lower salivary pH and differences in Prevotella abundance, but further research is required to confirm these observations. Notably, no studies were found assessing DPs such as ketogenic, low-FODMAP, or traditional Asian diets in relation to oral microbiota, revealing a clear gap in the literature. Ten studies employed interventions with prebiotic or probiotic supplementation. These studies were more consistent in design, predominantly randomized controlled trials, and reported beneficial effects such as increased levels of *Lactobacillus* and *Bifidobacterium*, improved microbial balance, and reduced presence of pathogenic taxa. However, variations in supplement strains, dosages, and durations limit direct comparisons. Despite promising trends, the heterogeneity in microbiome analysis techniques (e.g. 16S rRNA sequencing vs. culture- based methods), sampling methods (saliva, plaque, tongue), and populations (healthy vs. clinical) makes meta-analytic synthesis challenging. Additionally, most studies employed short-term interventions and lacked longitudinal follow-up. Overall, the current evidence supports a role for diet, both in terms of general patterns and targeted supplementation, in modulating oral microbial ecology. Moreover, oral hygiene products such as antimicrobial toothpastes and mouthwashes have been shown to alter the composition and function of the oral microbiome. Although not dietary, these interventions can affect biofilm dynamics and microbial diversity, potentially modulating or confounding the effects attributed to diet alone. Future studies should aim to standardize methodologies, explore underrepresented dietary models, and evaluate long-term clinical outcomes. Understanding how dietary modulation of the oral microbiome translates into preventive or therapeutic strategies remains an essential avenue for future research.

## Conclusions

A healthy lifestyle, characterized by a high intake of unprocessed foods and minimal consumption of refined sugars, exerts a significant influence on overall human health, including oral health. In general, DPs associated with traditional practices or informed nutritional decisions share common features that contribute to the prevention of oral diseases. Compared to the Western dietary model, these patterns are typically richer in plant-based foods and provide higher levels of bioactive compounds such as polyphenols, anthocyanins, and antioxidants. The intake of natural compounds has been shown to modulate the composition and function of the oral microbiota, potentially resulting in beneficial outcomes for individuals with oral diseases, particularly dental caries and periodontal conditions. Further, an increasing body of research has been endorsing the treatment effectiveness of probiotics and prebiotics supplementation for the management of oral diseases. This review presents the latest advancements in scientific literature regarding the impact of diet and nutrition on oral health. We consider that nutritional intervention strategies should be established based on healthier foods, including supplementation with probiotics and prebiotic compounds to increase protective bacterial species in the oral cavity.

## Data Availability

The data used to support the findings of this study are included within the article. Additional information can be requested by contacting the corresponding author.
